# A personalized tutorial to improve understanding of individual chemical results and opportunities for reducing exposure

**DOI:** 10.1038/s41370-026-00840-3

**Published:** 2026-02-07

**Authors:** Katherine E. Boronow, Aaron Maruzzo, Rachel A. Morello-Frosch, Anisha Nakagawa Patil, Erin DeMicco, Phil Brown, Amy M. Padula, Sarah D. Geiger, Julia Green Brody

**Affiliations:** 1 Silent Spring Institute, Newton, MA USA; 2 School of Public Health and Department of Environmental Science, Policy and Management, University of California, Berkeley, Berkeley, CA USA; 3 Conservation Law Foundation, Boston, MA USA; 4 Program for Reproductive Health and the Environment, Department of Obstetrics, Gynecology and Reproductive Sciences, University of California, San Francisco, San Francisco, CA USA; 5 Social Science Environmental Health Research Institute, Northeastern University, Boston, MA USA; 6 Department of Health and Kinesiology and Beckman Institute for Advanced Science and Technology, University of Illinois at Urbana-Champaign, Champaign, IL USA

**Keywords:** Environmental health literacy, Return of results, Science communication, Environmental chemicals, Graph literacy

## Abstract

**Background:**

Environmental health studies frequently measure levels of harmful chemicals in people or personal spaces, and returning those individual levels is an ethical responsibility and important opportunity to teach people about chemical exposures and how to reduce them.

**Objective:**

We sought to enhance meaningful report-back by quantitatively evaluating a personalized tutorial designed to support environmental health literacy about personal chemical exposures.

**Methods:**

We developed a novel smartphone-based tutorial that used the Predict-Observe-Explain educational framework to increase understanding of personal results graphs and promote taking actions to lower exposure. We deployed the tutorial as part of report-back in the Illinois Kids Development Study and Chemicals in Our Bodies pregnancy cohorts, and we collected digital analytics on how participants (*n* = 295) interacted with it. We tested the effect of the tutorial on participants’ accuracy at answering four graph-reading questions and examined differences by educational attainment and socioeconomic status. The tutorial prompted participants to select exposure sources that were relevant to them, and we calculated response frequencies of participants’ self-reported interest in taking related actions.

**Results:**

A total of 92% of participants (*n* = 270) completed the Predict and Observe phases of the tutorial. Among those participants, 70% (*n* = 188) correctly answered all four graph-reading questions on their first attempt (without tutorial assistance), and success increased to 96% (*n* = 258) after the tutorial provided feedback and participants could make a second attempt. Improvement was greatest among participants without a bachelor’s degree. Participants who answered the Explain phase (*n* = 182) expressed high interest in trying new behaviors to reduce exposure.

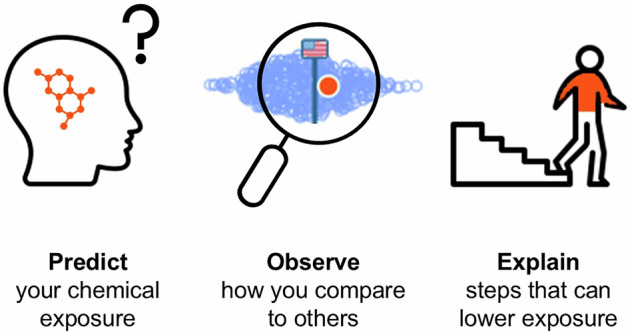

**Significance:**

While most participants understood their personal exposure graph without assistance, the tutorial successfully reduced differences in understanding by educational attainment. The tutorial was also effective at creating intentions to adopt health-protective behaviors. Scalable tools like this can support effective report-back in populations with all levels of environmental health literacy.

**Impact:**

To help people at all educational levels learn about their chemical levels and how to reduce them, we developed a personalized tutorial for use in reporting back results in environmental exposure studies. Before tutorial assistance, participants without a bachelor’s degree had lower understanding of their personal results graphs. Tutorial assistance successfully reduced differences in understanding between those with and without a bachelor’s degree. In addition, the tutorial was effective at creating intentions to adopt health-protective behaviors based on personalized recommendations for each participant. This scalable digital tool supports understanding and action during report-back of chemical exposure results.

## Introduction

Environmental health studies often measure personal chemical exposures, for instance, in people’s blood, urine, or household dust or air, posing responsibilities for researchers to communicate with participants about their own results, a practice called report-back. While results for certain chemicals, such as lead, are routinely reported back using clinical guidelines, studies often include contaminants of concern for which science does not yet support specific action levels, including, for example, flame retardants, plasticizers, per- and polyfluoroalkyl substances (PFAS), and compounds in personal care products. Participants nearly always want to know their own results even when health effects are uncertain [1, 2], and expert consensus recognizes that reporting back is an ethical duty and offers benefits for participants and researchers [2]. For many emerging contaminants, experts agree that evidence for health effects, though incomplete, is strong enough to recommend limiting future exposures [3–5], so report-back is an important opportunity to educate study participants and motivate behavior changes [6–12]. In this study, we sought to advance methods to communicate with study participants about their personal chemical levels and ways to reduce them.

To benefit participants, results must be returned in an information-rich and accessible context. Interviews have shown that people want to know what was measured, where it comes from, how much was detected, whether measured levels are concerning, and what they can do [13]. We previously designed personal exposure reports that answer these questions using text, graphs, and images, and we developed the Digital Exposure Report-Back Interface (DERBI) as a web application for preparing them at scale [14]. DERBI reports have been shown to benefit participants’ environmental health literacy (EHL) about chemical exposures, which encompasses both their understanding of the risk and how to mitigate it [15]. After report-back, participants had greater concern about the health effects of chemicals in consumer products and took some exposure-reducing actions [6], but additional opportunities remain to further support participant understanding and action. In addition to providing brief text summaries of notable personal results, DERBI reports use graphs to more completely represent quantitative results. Well-designed graphs can leverage people’s natural visual abilities to make greater than and less than comparisons [16], so they are suited to questions of “how much?”, “is that high?”, and “is that safe?” Because results graphs can quickly communicate about what was found and potential for exposure reduction, their interpretability is integral to the success of report-back. Thus, we targeted the results graphs as a key locus for building EHL among study participants.

**Fig. 1:**
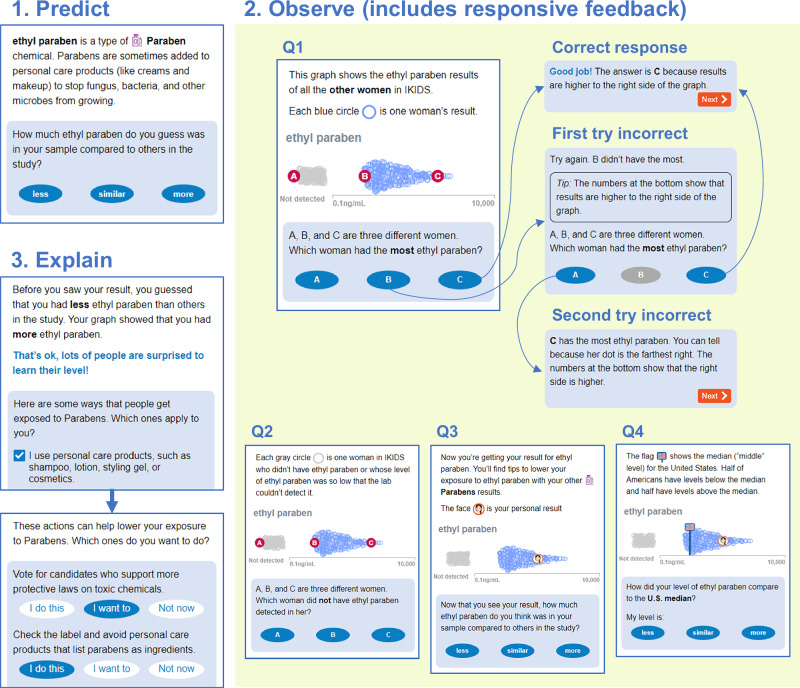
Screenshots from the graph-reading tutorial showing the Predict, Observe, and Explain phases (clockwise). During the Observe phase, participants answered four graph-reading questions (Q1–Q4). An example of the responsive feedback that participants received after each question is shown for Q1. Not all screens in the tutorial are shown.

Environmental exposure data requires different visualization approaches than typical models for health communication, which focus on conveying probabilistic risk (i.e., risk of developing a disease) [17] or showing medical test results with clinical guidelines (i.e., is a health biomarker within a standard range) [18]. In contrast, for emerging contaminants, scientific knowledge cannot yet quantify health risks or define levels below which exposures are not expected to affect health. In this context, many environmental health studies use strip plots to visually communicate useful information about exposure levels. Strip plots show measured chemical levels in a population as data points along an axis of concentration level. Sina plots are a variant where the relative density of the distribution is plotted in a second dimension to create a silhouette like a violin plot (the style of sina plot used in this study can be seen in Fig. 1). These plots convey information about range, central tendency, and variability in the study distribution, and they can also show the frequency of non-detects (measurements below the level that the laboratory can accurately quantify). By highlighting an individual’s result on the overall distribution, participants have the opportunity to compare their own exposure level to others in the study. In our experience, participants intuitively want to have lower levels of chemicals measured in their sample. Showing reference data from other sources can enable additional comparisons, for example, to U.S. nationally representative estimates from the National Health and Nutrition Examination Survey (NHANES) or government guidelines, such as drinking water standards.

To be equitable, graphs should be accessible to participants of all levels of scientific literacy and numeracy [19, 20]. Available evidence is limited but suggests that strip plots are an effective option, as summarized below. We have observed participants with and without college degrees reading strip plots during one-on-one usability testing, which is conducted as part of the report development process to get feedback on all aspects of the report. Usability observations show that although some people initially lack confidence and hesitate to begin reading the graphs, most ultimately succeed [21]. In the Child Health and Development Studies, 15 of 18 participants responded positively in qualitative interviews about how results were presented in a report using strip plots [14]. In focus groups with 31 participants in the Houston Hurricane Harvey Health study, participants preferred strip plots rather than bar charts with error bars [22], and in a New York City study of air pollutants, fewer than 20% of participants said it was “difficult” to find their levels in a report using strip plots [23]. Other graph formats have recognized limitations. Viewers commonly misinterpret boxplots [24], and bar charts show only one piece of information from a distribution (such as the median) and no information about range or variability. Additionally, people are likely to underestimate others’ exposures based on bar charts showing central tendency, because values below the top of the bar are perceived as more likely than values above it [25]. Different graphs are effective at highlighting guideline comparisons, such as a number line with shaded regions [20], but emerging contaminants frequently lack such guidelines.

Although evidence supports the use of strip plots, some participants may initially be less comfortable with them. Prior printed reports have sometimes included a “how to read your graph” figure [21], but digital report-back offers additional opportunities for multi-stage learning. Thus, we built an interactive graph-reading tutorial within a smartphone-based results report. The dual EHL goals of the tutorial were to support participants’ confidence and success in understanding their personal exposure results and to guide strategic decision-making about how to reduce them. The tutorial was designed using the Predict-Observe-Explain (POE) educational framework, a validated learning strategy used in science education. The learner first predicts the outcome of a scenario, then observes the actual outcome (for example, through experiment or demonstration), and finally explains what they observed in relation to their prediction [26]. Our POE-style graph-reading tutorial was designed to advance multiple learning objectives: (1) stimulate participants’ curiosity about their personal exposure results (Predict phase); (2) build participants’ confidence in interpreting results graphs and develop graph-reading skills among those needing greater support (Observe phase); and (3) connect participants’ personal behaviors to the chemical levels in their bodies and encourage them to try new behaviors (Explain phase). These learning objectives align with progressive stages of environmental health literacy by helping people recognize and understand their personal exposure, analyze where it might come from, and finally translate that knowledge into action to reduce exposure [27]. The tutorial goals extend beyond graph recognition to equipping users with a framework for prioritizing actions based on personal exposure sources and then setting intentions to act on them.

We describe here the development of the POE-style tutorial and its experimental deployment in two pregnancy cohorts returning chemical exposure results. We hypothesized that most participants would be able to read the strip plots without any guidance, because they are designed to rely on visual comparisons rather than formal graph literacy. However, some research has suggested that graph literacy for other visualization types (line plot, bar chart, pie chart, and icon array) may be influenced by educational attainment and socioeconomic status (SES) [28, 29], so we investigated whether these factors influenced understanding of our strip plots. We hypothesized that the tutorial would build graph-reading skills among those with lower baseline understanding and thereby reduce any differences in graph literacy due to formal education or SES. This study tests the ability of a brief, interactive educational intervention to enhance the experience of study participants receiving their own chemical exposure results by supporting graph-reading and connecting personal results with opportunities for exposure reduction.

## Methods

### Study populations

This study was conducted in two prospective birth cohorts within the National Institutes of Health Environmental Influences on Child Health Outcomes (ECHO) consortium. The Illinois Kids Development Study (IKIDS), based in Champaign-Urbana, Illinois, and the Chemicals in Our Bodies (CIOB) study, based in San Francisco, California, are jointly examining the effects of chemical and psychosocial stressors during pregnancy on child neurodevelopment. Recruitment and eligibility details are reported elsewhere [30].

Participants eligible for this report-back study contributed biological samples during pregnancy and had chemical measurements available in 2020 when the personal exposure reports were prepared. In CIOB, eligible participants expressed interest in receiving personal results as part of the informed consent for the parent study. CIOB and IKIDS participants who enrolled in this report-back study consented to receive their personal results as part of the informed consent form that was approved by the institutional review boards at the University of California San Francisco (protocol 18-26810) and the University of Illinois Urbana-Champaign (protocol 09498), respectively.

CIOB participants received blood measurements of polybrominated diphenyl ether (PBDE) flame retardants and/or PFAS, and IKIDS participants received urine measurements of 11 phenolic compounds. No participants had received their results previously. Participants were English speakers in IKIDS, and English and Spanish speakers in CIOB. All study materials (including informed consent, email communications, results reports, and data collection instruments) were professionally translated into Spanish and reviewed by bilingual members of the research team. No reverse translation was required for the data analysis reported here.

After the conclusion of the study, eligible participants who did not participate in the study were invited to view their personal results report.

### Study design

We experimentally tested two independent interventions to enhance report-back, a POE-based graph-reading tutorial within the personal exposure report (described below) and follow-up emails that encouraged viewing the report and taking action to lower exposures. Participants were randomized to receive no intervention (standard report-back), tutorial only, email only, or both tutorial and email. We report here on participants randomized to receive the tutorial.

Participants were recruited to the report-back study by email and received up to five invitations at 5-day intervals. The invitation informed participants that their personal results were available and asked them, after informed consent, to complete a 10-min online pre-survey to view their results. After the pre-survey, participants immediately received login information to access their personal exposure report. Participants who opened their report were invited by email 2 weeks later (up to five invitations at 5-day intervals) to complete a 20-min post-survey and received a gift card for completing all study activities ($10 in IKIDS and $20 in CIOB, the differing amounts reflect geographic differences in cost of living). Participants were not eligible for the post-survey until they viewed their personal report, and participants received at least one reminder email to open their report.

### Personal results reports

The personal exposure reports were produced using DERBI [14] and included background information about the chemicals measured in the study—including sources, how they might affect health, and actions for reducing exposure—as well as overall study results, strategies for community action, and answers to common questions. Report content was prepared by the research team. Individual results were depicted in sina plots showing the participant’s level relative to the study distribution and U.S. median for reproductive-aged women (18–40 years) in NHANES. Most of the U.S. medians were calculated from the 2015–2016 NHANES cycle (for phenolic compounds, PBDEs, and eight PFAS); four PFAS were included from 2013–2014 or 2011–2012 when they were last measured. The report was optimized for smartphone and could also be viewed on a tablet or computer. We recorded participant interactions with the online report using digital analytics. Print reports were provided upon request.

### Graph-reading tutorial

The graph-reading tutorial was embedded within the DERBI report and was developed de novo for this study, using the POE framework, to address our objectives of building participants’ confidence in reading the results graphs and supporting exposure reduction. Author KEB developed the logic flow, and author ANP developed the graphic designs. To improve the design of the tutorial, ANP conducted a formative evaluation of the tutorial prototype with a convenience sample of 10 individuals. The prototype was tested using an interactive digital mock-up in InVision, a UX design software that allowed a user to interact with the tutorial as simulated for one person. The evaluation protocol was described to potential testers, and usability tests were conducted with those who agreed. Testers were asked to think out loud while navigating the mock-up for the first time to elicit their raw reactions and impressions. The researcher did not provide feedback or answer questions during this phase to avoid influencing the testers. After completing the tutorial, the researcher asked brief follow-up questions such as how confident the tester felt after completing the tutorial and if there was anything that was still confusing about the graph. Feedback from usability testing was analyzed qualitatively (organized according to points of difficulty, differences in expectations, likes, suggestions, questions, and wordings), and design changes were implemented. The final design was then created with software and integrated into the DERBI smartphone report developed for this study by ANP.

The graph-reading tutorial, depicted in Fig. 1, was automated to use an actual chemical biomonitoring result for each participant. During the Predict phase, the tutorial described how people usually get exposed to the featured chemical. Then the participant was asked to guess how much of the chemical was in their sample compared to others in the study (less, similar, or more). The goal of the Predict phase was to generate curiosity about their personal exposure, guided by information about where the chemical comes from.

During the Observe phase, the participant answered four graph-reading questions. Each question displayed the actual study distribution for the chemical. The first two questions addressed interpretation of the x-axis: three fictional data points were overlaid on the distribution and the participant was asked to choose the point with the highest level of the chemical and the point that had no chemical detected. For the second set of questions, the participant’s actual personal result was plotted on the study distribution and participants were asked to make comparisons. First, the participant was asked how their result compared to others in the study. Then, the U.S. median was added, and the participant was asked how their result compared to that level. The avatar used to represent the participant’s personal result was selected by the participant earlier during the login process. Participants received responsive feedback for every answer. After a correct response, the tutorial gave positive reinforcement and a short explanation of why the answer was correct. After an incorrect response, the tutorial offered a tip about how to read the graph and asked the participant to try again. After a second incorrect response to the same question, the tutorial gave a longer explanation. At the end of the Observe phase, the participant was instructed how to expand the graph to see their exact level (as in the real report) and was shown an explanation of the units of measure. The goal of the Observe phase was to build participants’ confidence in interpreting results graphs or help strengthen their graph-reading skills as needed.

The Explain phase began with a comparison between the participant’s prediction of their relative chemical level and how their level actually compared to others in the study. Then, the participant completed a checklist indicating whether they were exposed to common sources of the chemical. In the final step, the tutorial returned a customized list of exposure reduction actions based on their indicated sources. For each action, the participant could select “I do this,” “I want to,” or “Not now.” The goal of the Explain phase was to help participants understand how their behavior affects exposure and support them in taking personally-relevant exposure reduction actions.

After completing the tutorial, participants saw options to view all their chemical results or to retake the tutorial. The option to view all results was highlighted for participants who answered all four questions correctly; the option to retake the tutorial was highlighted for participants who got any question wrong after two tries. If after taking the tutorial a second time they got more than one question wrong, the participant was encouraged to contact the research team for assistance. Participants could exit the tutorial at any time.

As noted, participants received real personal chemical results during the tutorial. Logical criteria were applied for selecting the specific chemicals shown to each participant to ensure the tutorial displayed a distribution with all highlighted graph elements. The tutorial always started with the chemical result with the highest percentile rank meeting as many criteria as possible, and if a participant proceeded to immediately retake the tutorial, the decision rules picked the chemical with the lowest percentile rank meeting as many criteria as possible. Sources and exposure reduction actions shown in the Explain phase were based on the chemical group of the selected chemical and therefore differed between participants.

Participant activity on the tutorial was collected with digital analytics that recorded each user event with a timestamp.

### Demographic variables

We categorized educational attainment as “no bachelor’s degree” (less than high school, high school diploma or GED, some college, technical school, or associate degree) or “bachelor’s degree or more” (bachelor’s, master’s, or doctoral degree). We defined SES based on the combination of health insurance status and household income to reduce the impact of missing data and to adjust for state-specific differences in income. SES was categorized as “low” if a participant reported having public health insurance with income restrictions (Medicaid or Medi-Cal) or household income less than the median in their state (<$80,000 for CIOB and <$70,000 for IKIDS); “high” SES participants did not meet either “low” criterion and were not missing data for both variables.

### Data analysis

We restricted the analysis to participants randomized to the graph-reading tutorial who accessed their online report. We expected participants to use the tutorial before viewing other personal results because that was the prominent navigation path. We excluded participants who instead viewed other chemical results before the tutorial, to ensure tutorial results reflected participants’ first time seeing the results graphs. Two participants missing SES and/or education data were excluded from analyses using those variables. We included analytics data recorded within 180 days of completing the pre-survey and processed it to adjust for technical inconsistencies (e.g., duplicate page loads). Data analysis was performed in R (version 4.2.2; R Development Core Team).

Because SES and education did not differ by cohort (Fisher’s exact test, Table S1), we combined data from CIOB and IKIDS for analysis.

#### Tutorial use

We analyzed how many times participants started the tutorial and how far they got. Using differences between timestamps, we calculated the time spent on the tutorial for each participant (overall and for specific activities). When 10 min or more elapsed between actions, the user was assumed to be inactive, and inactive time was excluded. For the Predict, Observe, and Explain analyses, we analyzed responses from the first time a participant accessed the tutorial until they completed it, skipped it, or viewed a chemical result page.

#### Predict phase

We compared participants’ first predictions of their relative chemical exposure level to their actual relative exposure level categorized by percentiles in the study distribution: less (≤25^th^), similar (>25^th^ to ≤75^th^), and more (>75^th^). We examined the overall distributions and then stratified by the chemical group featured in the tutorial.

#### Observe phase

Responses to the four graph-reading questions were categorized as correct on the first try, correct on the second try (after an incorrect answer), incorrect after two tries, or exited. We defined “exited” as the point when a participant stopped engaging with the tutorial. We visualized responses of participants who reached the first question and calculated response frequencies among participants who answered all four questions.

We calculated the number of correct responses for each participant based on their first response only and based on both their first and second tries (range = 0–4). We used first responses to evaluate effectiveness of the graphs without the tutorial. To evaluate the effectiveness of the tutorial, we used Wilcoxon signed-rank tests (alpha = 0.05) to examine differences in total correct responses at baseline (first try only) and after tutorial feedback (first and second tries combined), first among all participants and then stratified separately by education or SES. We used Wilcoxon rank-sum tests to test for differences between subgroups in the total correct responses at baseline and after tutorial feedback.

#### Explain phase

We used Fisher’s exact test to assess differences in education and SES among participants who responded to the Explain phase and those who completed the Observe phase but did not respond to the Explain phase. We calculated response frequencies for exposure reduction actions that had at least 20 responses. To assess which actions had the highest potential uptake, we calculated the percentage of “I want to” responses among participants who did not already perform the action.

#### Satisfaction

We analyzed response frequencies to the post-survey question about satisfaction with the tutorial.

## Results

### Participation

Within the larger report-back study, 310 participants (*n* = 139 CIOB, 171 IKIDS) assigned to the graph-reading tutorial opened their report and were eligible for this analysis. Seven (*n* = 4 CIOB, 3 IKIDS) were excluded for visiting a chemical results page before the tutorial, and eight (*n* = 2 CIOB, 6 IKIDS) were excluded for never opening the tutorial, resulting in a sample of 295 participants (*n* = 133 CIOB, 162 IKIDS).

Education and SES did not significantly differ between IKIDS and CIOB (Table S1). Overall, 84% of participants (*n* = 247) had a bachelor’s degree or more, 71% (*n* = 209) were categorized as high SES, and 98% (*n* = 290) were English speakers (Table 1). A total of 67% (*n* = 197) self-reported as non-Hispanic white (Table 1), and CIOB had a significantly greater proportion of participants who identified as Hispanic or a person of color compared to IKIDS (Table S1).

**Table 1:** Characteristics of participants in the CIOB and IKIDS cohorts who opened the tutorial before viewing a chemical results page (*n* = 295).

Demographic category	Response level	*n*	(%)
Race/ethnicity	Non-Hispanic White	197	(67)
	Person of color or Hispanic^a^	97	(33)
	Missing	1	(<1)
Education	Bachelor’s degree	247	(84)
	No bachelor’s degree	46	(16)
	Missing	2	(<1)
Health insurance	Not insured	2	(<1)
	Medi-Cal or All Kids (Medicaid)	41	(14)
	Private or other insurance	241	(82)
	Missing	11	(4)
Household income	$0–19,999	13	(4)
	$20,000–39,999	24	(8)
	$40,000–$79,999	59	(20)
	$80,000 and over	195	(66)
	Missing	4	(1)
Socioeconomic status (SES)^b^	High SES	209	(71)
	Low SES	84	(28)
	Missing	2	(<1)
Language	English	290	(98)
	Spanish	5	(2)

^a^Ten participants self-reported as non-Hispanic Black, 48 as non-Hispanic Asian, 1 as non-Hispanic Native Hawaiian or other Pacific Islander, 9 as non-Hispanic and more than one race, and 29 as Hispanic.

^b^Lower SES participants were defined as having Medicaid/Medi-Cal insurance status or household income less than the state median (<$80,000 CIOB or <$70,000 IKIDS). Higher SES participants did not meet either criterion for lower SES and were not missing data for both variables.

### Tutorial use

Most participants started the tutorial only one time (*n* = 214, 73%), and 92% (*n* = 270) answered the Predict phase and the four Observe graph-reading questions. Of the 191 participants who completed the tutorial, 9% (*n* = 18) did so more than once. Among participants who exited the tutorial partway on their first use, most (64%, *n* = 68/106) left after the graph-reading questions and before the Explain phase.

Participants spent 2.6 (1.8–3.8) min (median and interquartile range) on the tutorial across all their report activity. Among 189 participants who completed the entire tutorial on their first use, the median completion time was 2.8 (2.3–4.0) min. Among participants who completed the four graph-reading questions on their first use (*n* = 264), the median time to complete those questions was 1.4 (1.1–1.8) min.

### Predict phase

**Fig. 2:**
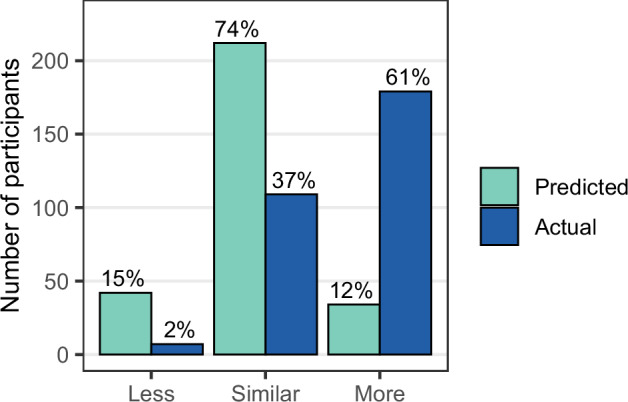
Participant guesses of their relative exposure during the Predict phase of the tutorial and their actual relative chemical level based on percentile relative to the study distribution: less (≤25th), similar (>25th to ≤75th), and more (>75th). The chemical shown in the tutorial was selected for each person based on several criteria; if available, participants preferentially saw a chemical having a relatively high exposure level.

Two hundred eighty-eight participants made a prediction. Most (*n* = 212, 74%) guessed their result would be similar to others, which often underestimated their actual exposure since the tutorial preferentially first selected a chemical with a relatively high exposure (Fig. 2). One hundred nine (38%) participants guessed correctly. Chemicals featured in the tutorial belonged to one of five chemical groups (antimicrobials, bisphenols, parabens, PBDEs, or PFAS), and the trend of underestimating personal exposure was consistent regardless of the chemical group shown (Fig. S1). However, the antimicrobial group had the highest proportion of “more” guesses (24%) and the highest rate of correct guesses (58%).

### Observe phase

To examine how well participants could interpret the graphs without help from the tutorial, we calculated response frequencies for first attempts at answering the graph-reading questions. One hundred eighty-eight (70%) correctly answered all four questions on their first attempt, and an additional 58 (21%) got three correct (Table S2).

**Fig. 3:**
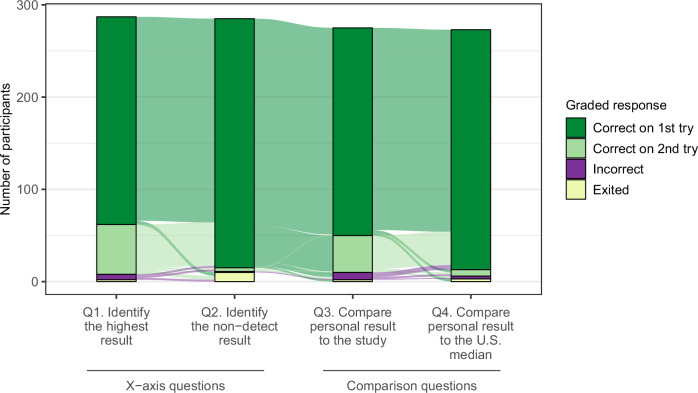
Graded participant responses to four graph-reading questions during the Observe phase of the tutorial. Shaded areas between bars represent participant transitions between questions and response types.

To evaluate whether the tutorial improved graph-reading, we used an alluvial diagram to visualize first and second responses across the four questions among participants who reached the first graph-reading question (*n* = 287) (Fig. 3). Because the Observe phase included two pairs of conceptually related questions, information gained by answering the first question of the pair (Q1 and Q3) may help to answer the second question of the pair (Q2 and Q4). Among participants who answered all four questions, more answered the second question in each pair correctly on the first try: 81% correct Q1 vs. 98% Q2 for the *x*-axis questions, and 82% Q3 vs. 96% Q4 for the comparison questions (Table S3). We also observed that nearly all participants who took two tries on Q1 or Q3 were correct on their first try for Q2 or Q4 (Fig. 3, light green flows between question pairs).

**Fig. 4:**
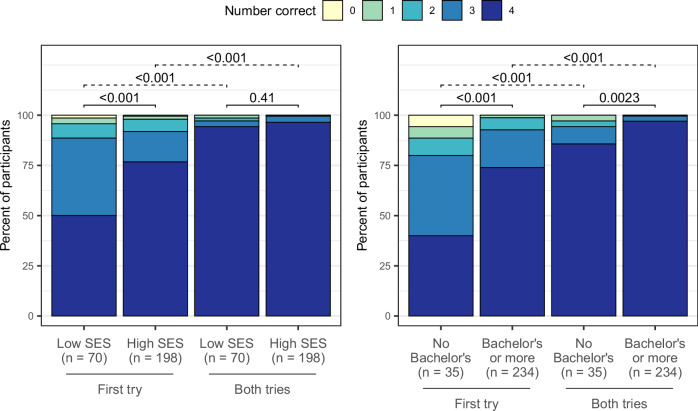
Frequencies of graph-reading tutorial scores among participants who answered all four graph-reading questions, stratified by SES (*n* = 268) and education status (*n* = 269). Wilcoxon rank-sum tests for unpaired samples (solid lines) were used to assess score differences by SES and education subgroups. Wilcoxon signed-rank tests for paired samples (dashed lines) were used to assess change in scores within each subgroup between tries. Summary data are available in Table S2.

Next, we considered improvement between tries. Overall, participants answered significantly more questions correctly on their first and second tries combined compared to their first try alone (Wilcoxon signed-rank test, *p* < 0.001). This pattern persisted when separately stratifying by education and SES (Fig. 4). We saw the largest improvement—a 46-percentage point increase in answering all four questions correctly—among participants without a bachelor’s degree (Fig. 4 and Table S2).

Finally, we assessed differences by SES and education. Participants with less formal education or lower SES answered significantly fewer questions correctly on their first try (Fig. 4). After tutorial assistance, the number of correct responses no longer significantly differed by SES (Fig. 4). Number correct remained significantly lower among those with less formal education after tutorial assistance (Fig. 4); however, the difference between those with and without a Bachelor’s degree decreased dramatically. On the first try, there was a 34-percentage point difference in the proportion of participants getting all four correct between those with and without a Bachelor’s degree, and after tutorial assistance the gap narrowed to 11 points (Table S2).

For each graph-reading question, the proportion of participants giving an incorrect response after two attempts ranged from 0.4% to 3.0% (*n* = 1–8, Table S3), and 99% of participants (*n* = 267) correctly answered at least three questions correctly on their first and second tries (Table S2).

### Explain phase

**Fig. 5:**
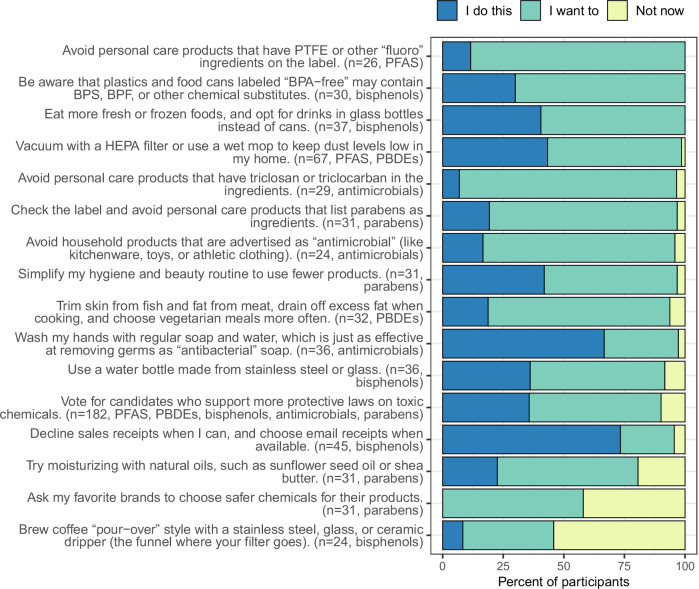
Distribution of responses to exposure reduction actions among participants who answered the Explain phase of the tutorial (*n* = 182). Exposure reduction actions are ordered from highest to lowest actionability (percentage of “I want to” responses among participants who did not already do the behavior). Participants saw different actions based on the chemical shown in the tutorial and the personal exposure sources they selected, and the relevant chemical groups are labeled for each action.

One hundred eighty-two participants indicated their interest in taking exposure reduction action. Participants who responded to the Explain phase did not significantly differ by education or SES from participants who completed the Observe but not the Explain phase (Table S4). Each participant received a personalized list of exposure reduction tips (2 to 6 tips per participant) based on the featured chemical and the exposure sources they selected. Across all participants, 24 unique exposure reduction tips were shown in the Explain phase, and 16 met the criterion for analysis ( ≥ 20 responses). The actions that participants most frequently said they “already do” were declining sales receipts (*n* = 33/45, 73%) and washing hands (*n* = 24/36, 67%) (Fig. 5). Participants were interested in trying new behaviors: for 14/16 actions, 75% or more participants said they “want to” try it, among those not already doing it (Fig. 5). Among the 16 actions, 3 to 5 were relevant to each chemical group (including one community-level action relevant to all groups).

### Satisfaction

Out of 175 participants who answered the post-survey question, “The introductory graph-reading exercise helped me understand my results,” 110 (63%) strongly agreed, 59 (34%) somewhat agreed, 5 (3%) somewhat disagreed, and 1 (<1%) strongly disagreed. Of the 6 who disagreed, 5 got all four graph-reading questions correct on their first tries and 1 exited at the Predict phase.

## Discussion

Returning individual exposure results for emerging contaminants requires communications approaches that differ from traditional health messaging. To help people understand and engage with their personal chemical results, we built a novel digital tutorial following the predict-observe-explain educational framework. The tutorial was successful at growing participants’ environmental health literacy by supporting understanding of personal results graphs and prompting interest in trying new behaviors to reduce chemical exposures.

As expected and consistent with the intent of the graph design and previous usability testing, most participants could interpret their results graphs without tutorial assistance: 70% correctly answered all four questions on the first try, underscoring the readability of the strip plot format. The tutorial increased understanding among those who did need guidance, showing its value for advancing equity. The tutorial produced the largest improvement in understanding of personal chemical exposures among participants with less formal education, a result with important implications for communities facing disproportionate exposure burdens and often with fewer resources to learn about environmental health. With tutorial assistance, 99% of participants correctly answered at least 3 out of 4 graph-reading questions. Smartphone-compatible reports increase access to digital report-back tools, because people who rely on smartphones for internet tend to have lower household incomes and less formal education [31], and the graph-reading tutorial further supports our accessibility goals.

Participants who reached the Explain phase were very interested in trialing new exposure-reducing behaviors. The actions highlighted in the Explain phase were personalized for each participant based on self-reported exposure sources, which likely contributed to high willingness to try. Additionally, the tutorial provided the participant’s personal result for the selected chemical, and previous work has shown that report-back generally can motivate action to reduce exposures [6, 8, 9]. Because of the tutorial design, we cannot isolate the effect of the Explain phase on participant motivations independently from learning a personal result. We expected that participants could apply the framework modeled by the tutorial—prioritizing actions based on personally-relevant chemical sources—to other chemicals in their report, although we had no way to assess this. The tutorial also highlighted the disparity between people’s naïve expectations of their personal exposure (typically, people expected their level to be similar to others) and their actual exposure, and we hypothesize that this discrepancy may have been motivating and contributed to interest in trying new behaviors. In the integrated behavioral model, intention to perform a behavior is the most important predictor of whether a person does so, although other factors also affect uptake [32]. Participants’ high interest in trying behaviors identified in the tutorial suggests that the tutorial succeeded in generating intentions, but we did not evaluate whether participants acted on these intentions. Longer-term adoption of behavior is an important topic for future study.

The tutorial highlighted a chemical from one of five chemical groups that differ, for example, in their sources and how quickly they are eliminated from people’s bodies, but we did not anticipate or observe major effects on tutorial outcomes by chemical group. In the Predict phase, the tendency for people to expect that their exposure was similar to others was seen across all the chemical groups. This suggests that self-assessing relative exposure based on brief source information was difficult, likely because these chemicals are all found in products that most people use regularly and are not clearly labeled. Somewhat greater accuracy observed for antimicrobial chemicals is consistent with the fact that these chemicals are more “visible” to consumers through marketing statements that are used for decision-making. We did not examine differences by chemical group in the Observe phase, because the graph-reading tasks were independent of the chemical group. In the Explain phase, people’s perception of the difficulty of the suggested actions could potentially vary by chemical group, but we found that actions of high interest spanned all groups. The elimination rate varies widely among the chemical groups and could affect motivation to take action. Elimination rate is discussed elsewhere in the results reports, but that information was not provided within the tutorial context and so was not likely to influence the Explain results. Further investigating differences in participants’ responses to report-back for different types of chemicals is a valuable future direction.

A limitation of the tutorial was that many participants left after the final graph-reading question and before the Explain stage. We hypothesize that participants may have been eager to access more of their results, or the interface may not have indicated clearly enough how to proceed, so exiting appeared like the only option. We have observed that participants readily navigate to pages with more information about their own chemical results [33], so in the future, integrating the Explain phase into these pages will ensure that it is easy to access and available for all chemicals in the report, not just the one highlighted in the tutorial.

Although the tutorial focused on key graph-reading concepts, it did not address all nuances of displaying exposure data for emerging contaminants, and our study did not collect information on people’s understanding of these features. Showing the study distribution as a reference establishes a social context for interpreting a value that has little intuitive meaning [34]. The study distribution also effectively shows participants with higher levels that their exposure is potentially modifiable, since many other people have achieved lower levels. This is an important message because precautionary action to reduce exposure is recommended for many emerging contaminants even when health-based guidelines are not available [3–5]. However, participants can wrongly presume that the population norm provides information about risk and thus feel safer or more worried based on their relative exposure [11, 12, 34]. In fact, all exposures in the study might be above or below a risk-based threshold if one were known. Another interpretation challenge is that because environmental data usually span multiple magnitudes of concentration and have long tails at the upper range, studies typically use a logarithmic scale to make the distribution visible on the graph. However, participants may not realize how many times higher exposures are at the top of the range compared to the bottom. A future tutorial could address these features of the data and provide insight into understanding.

The tutorial had high acceptability among our cohort receiving results measured during pregnancy: it was accessed by nearly all participants, and 97% of those who used it agreed that it helped them to understand their results. The few who disagreed were already proficient at graph-reading, as indicated by their graph-reading scores. The tutorial was quick, taking about 3 min to complete. The study population was about one-third non-White and one-third lower SES, supporting the generalizability of our results, but the proportion of participants without a bachelor’s degree was relatively small. Additionally, low participation in the study among Spanish speakers prevented us from examining tutorial outcomes by language. We learned that email recruitment was not an effective strategy for reaching Spanish speakers in this cohort, and future research should use other recruitment strategies, such as text message or phone call. To further investigate the generalizability of our approach, the intervention should be tested again in different populations, including those with greater numbers of non-English speakers and people with lower levels of formal education.

In addition to providing personal results in a specific study, high-quality report-back has been shown to be a vehicle for adults’ free choice learning and can improve EHL by increasing understanding of chemical sources, effects on health, and opportunities for action [6, 11, 35]. The tutorial’s focus on graph literacy may additionally support EHL by improving data interpretation skills that are broadly transferable to other quantitative contexts and decision-making about personal and public health. For example, in a community science study of contaminants in harvested rainwater, the experience of sense-making for environmental data led participants to gain confidence in their ability to interpret data and graphs related to the COVID-19 pandemic and to see other public health applications for science [36].

As returning personal exposure results becomes more common, researchers need tools to support report-back in varied populations. Researchers have used strategies beyond written reports, including in-person group report-back meetings [9, 37] and interactive data physicalizations [38, 39]. In this study, we demonstrated a new digital approach—a tutorial using the POE educational theory—that is scalable to studies of all sizes and can be deployed regardless of whether a study is location-based. The brief tutorial successfully supported environmental health literacy about exposures to emerging chemical contaminants. It aided the interpretation of personal exposure graphs and reduced differences in understanding associated with educational attainment, and it created interest in trying new behaviors to lower exposures.

## Supplementary information

Supplementary information

## Data Availability

The dataset generated and analyzed during the current study is available in the figshare repository (10.6084/m9.figshare.30471899).
