# Supplementary material for: A personalized tutorial to improve understanding of individual chemical results and opportunities for reducing exposure
Source: J Expo Sci Environ Epidemiol. 2026 Feb 7;36(3):511–20. doi: 10.1038/s41370-026-00840-3 (PMC13143806; doi:10.1038/s41370-026-00840-3)

**Supplemental Information for**

A Personalized Tutorial to Improve Understanding of Individual Chemical Results and Opportunities for Reducing Exposure

Katherine E. Boronow,^1*^ Aaron Maruzzo,^1^ Rachel A. Morello-Frosch,^2^ Anisha Nakagawa Patil,^1,6^ Erin DeMicco,^3^ Phil Brown,^4^ Amy M. Padula,^3^ Sarah D. Geiger,^5^ Julia Green Brody^1^

^1^ Silent Spring Institute, Newton, MA, USA

^2^ School of Public Health and Department of Environmental Science, Policy and Management, University of California, Berkeley, Berkeley, California, USA

^3^ Program for Reproductive Health and the Environment, Department of Obstetrics, Gynecology and Reproductive Sciences, University of California, San Francisco, San Francisco, California, USA

^4^ Social Science Environmental Health Research Institute, Northeastern University, Boston, MA, USA

^5^ Department of Health and Kinesiology and Beckman Institute for Advanced Science and Technology, University of Illinois at Urbana-Champaign, Champaign, Illinois, USA

^6^ Conservation Law Foundation, Boston, Massachusetts, USA

*Corresponding author: boronow@silentspring.org

**This file contains:**

Tables S1 to S4

Figure S1

**Table S1.** Characteristics of participants in the CIOB and IKIDS cohorts who were eligible for analysis (n = 295), stratified by cohort status.

|  |  | **n (%)** | | | |  |
| --- | --- | --- | --- | --- | --- | --- |
| **Demographic category** | **Response level** | **CIOB (n = 133)** | | **IKIDS (n = 162)** | | **p-value^a^** |
| Race/ethnicity | Non-Hispanic White | 73 | (55) | 124 | (77) | <0.001 |
|  | Person of color or Hispanic^b^ | 59 | (44) | 38 | (23) |  |
|  | Missing | 1 | (<1) | 0 | (0) |  |
| Education | Bachelor's degree | 106 | (80) | 141 | (87) | 0.2 |
|  | No bachelor's degree | 25 | (19) | 21 | (13) |  |
|  | Missing | 2 | (2) | 0 | (0) |  |
| Health insurance | Not insured | 2 | (2) | 0 | (0) | <0.001 |
|  | Medi-Cal, Medicaid, or All Kids | 33 | (25) | 8 | (5) |  |
|  | Other or not specified insurance | 87 | (65) | 154 | (95) |  |
|  | Missing | 11 | (8) | 0 | (0) |  |
| Household income | $0 - 19,999 | 9 | (7) | 4 | (2) | <0.001 |
|  | $20,000 - 39,999 | 10 | (8) | 14 | (9) |  |
|  | $40,000 - $79,999 | 14 | (11) | 45 | (28) |  |
|  | $80,000 and over | 97 | (73) | 98 | (60) |  |
|  | Missing | 3 | (2) | 1 | (<1) |  |
| Socioeconomic status (SES)^c^ | High SES | 94 | (71) | 115 | (71) | 1 |
|  | Low SES | 38 | (29) | 46 | (28) |  |
|  | Missing | 1 | (<1) | 1 | (<1) |  |
| Language | English | 128 | (96) | 162 | (100) | 0.02 |
|  | Spanish | 5 | (4) | 0 | (0) |  |

^a^ Fisher's exact test used to assess differences in demographics by cohort status.

^b^ In CIOB, 5 participants self-reported as non-Hispanic Black, 24 as non-Hispanic Asian, 1 as non-Hispanic Native Hawaiian or other Pacific Islander, 5 as non-Hispanic and more than one race, and 24 as Hispanic. In IKIDS, 5 participants self-reported as non-Hispanic Black, 24 as non-Hispanic Asian, 4 as non-Hispanic and more than one race, and 5 as Hispanic.

^c^ Lower SES participants were defined as having Medicaid/Medi-Cal insurance status or household income less than the state median (<$80,000 CIOB or <$70,000 IKIDS). Higher SES participants did not meet either criterion for lower SES and were not missing data for both variables.

**Table S2.** Frequencies of tutorial scores (number of questions answered correctly) among participants who answered all four graph-reading questions.

|  | | **n (%)** | | | | | | | | | |
| --- | --- | --- | --- | --- | --- | --- | --- | --- | --- | --- | --- |
|  | | **Tutorial Score – First try** | | | | | **Tutorial Score – Both tries** | | | | |
| **Demographic category** | **Level** | **0** | **1** | **2** | **3** | **4** | **0** | **1** | **2** | **3** | **4** |
| Overall (n = 270) | -- | 2 (<1) | 5 (2) | 17 (6) | 58 (21) | 188 (70) | 0 (0) | 1 (<1) | 2 (<1) | 9 (3) | 258 (96) |
| Socioeconomic status (SES)^a^ | Low SES (n = 70) | 1 (1) | 2 (3) | 5 (7) | 27 (39) | 35 (50) | 0 (0) | 1 (1) | 1 (1) | 2 (3) | 66 (94) |
|  | High SES (n = 198) | 1 (<1) | 3 (2) | 12 (6) | 30 (15) | 152 (77) | 0 (0) | 0 (0) | 1 (<1) | 6 (3) | 191 (96) |
| Education | No bachelor's (n = 35) | 2 (6) | 2 (6) | 3 (9) | 14 (40) | 14 (40) | 0 (0) | 1 (3) | 1 (3) | 3 (9) | 30 (86) |
|  | Bachelor's or more (n = 234) | 0 (0) | 3 (1) | 14 (6) | 44 (19) | 173 (74) | 0 (0) | 0 (0) | 1 (<1) | 6 (3) | 227 (97) |

^a^ Lower SES participants were defined as having Medicaid/Medi-Cal insurance status or household income less than the state median (<$80,000 CIOB or <$70,000 IKIDS). Higher SES participants did not meet either criterion for lower SES and were not missing data for both variables.

**Table S3.** Frequency of correct and incorrect responses among participants (n = 270) who answered all four graph-reading questions.

| **Question** | **Graded response** | **n (%)** |
| --- | --- | --- |
| Identify the highest result | Correct after first try | 218 (81) |
|  | Correct after both tries | 266 (99) |
|  | Incorrect | 4 (1) |
| Identify the non-detect result | Correct after first try | 265 (98) |
|  | Correct after both tries | 269 (100) |
|  | Incorrect | 1 (<1) |
| Compare personal result to the study | Correct after first try | 222 (82) |
|  | Correct after both tries | 262 (97) |
|  | Incorrect | 8 (3) |
| Compare personal result to U.S. median | Correct after first try | 260 (96) |
|  | Correct after both tries | 267 (99) |
|  | Incorrect | 3 (1) |

**Table S4.** Characteristics of participants who answered all four graph-reading questions (n = 270), stratified by whether they responded to the Explain phase of the tutorial.

|  | | **n (%)** | | | |  |
| --- | --- | --- | --- | --- | --- | --- |
| **Demographic category** | **Response level** | **Did not respond to Explain phase^a^ (n = 88)** | | **Responded to explain phase (n = 182)** | | **p-value^b^** |
| Education | Bachelor's degree | 73 | (83) | 161 | (88) | 0.18 |
|  | No bachelor's degree | 15 | (17) | 20 | (11) |  |
|  | Missing | 0 | (0) | 1 | (<1) |  |
| Socioeconomic status (SES) | High SES | 62 | (70) | 136 | (75) | 0.55 |
|  | Low SES | 25 | (28) | 45 | (25) |  |
|  | Missing | 1 | (1) | 1 | (<1) |  |

^a^ Includes 9 participants who submitted the Explain phase without providing any responses.

^b^ Fisher's exact test used to assess differences in demographics by Explain phase completion status.

**Figure S1.** Participant guesses of their relative exposure during the Predict phase of the tutorial, stratified by chemical group shown, and their actual relative chemical level based on percentile relative to the study distribution: less (≤25th), similar (>25th to ≤75th), and more (>75th). The chemical shown in the POE was selected for each person based on several criteria; if available, participants preferentially saw a chemical having a relatively high exposure level.


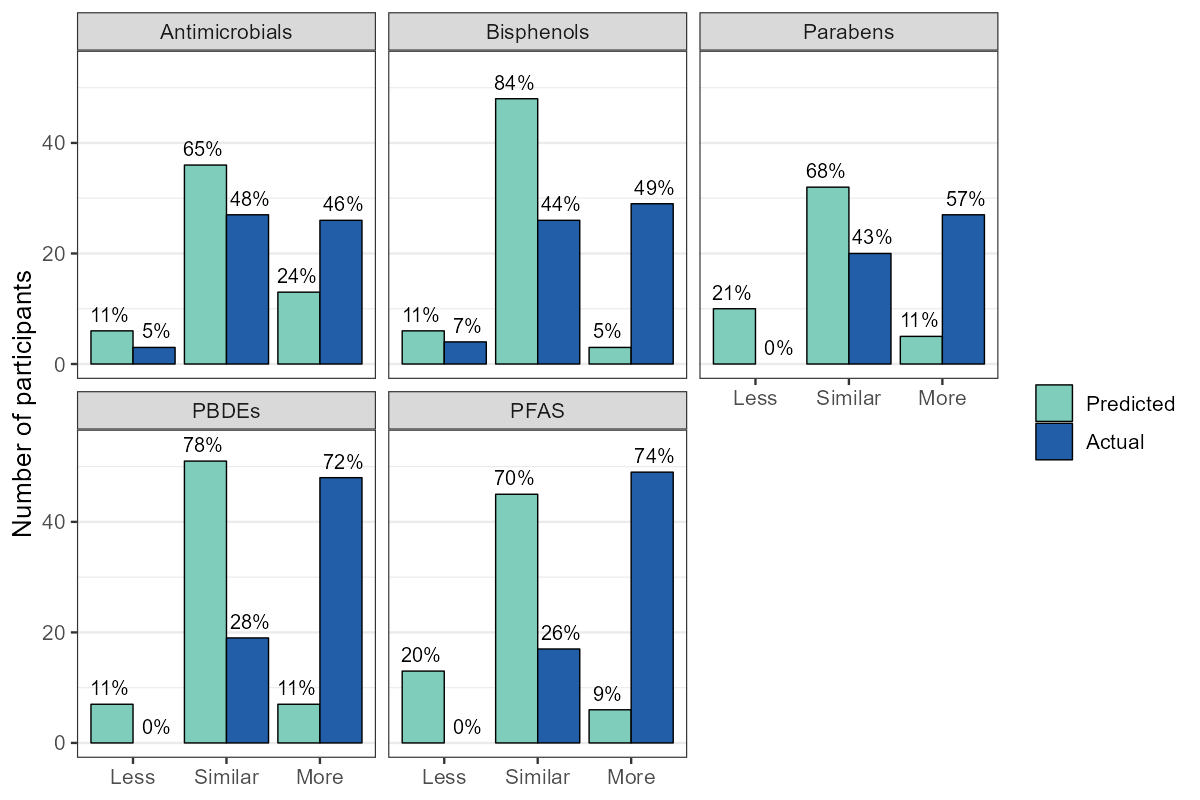

Supplement: Supplementary file 1 — Supplementary information [file 41370_2026_840_MOESM1_ESM.docx]
